# Short-term changes observed in multiparametric liver MRI following therapy with direct-acting antivirals in chronic hepatitis C virus patients

**DOI:** 10.1007/s00330-018-5788-1

**Published:** 2018-11-30

**Authors:** C. Bradley, R. A. Scott, E. Cox, N. Palaniyappan, B. J. Thomson, S. D. Ryder, W. L. Irving, G. P. Aithal, I. N. Guha, S. Francis

**Affiliations:** 1 Sir Peter Mansfield Imaging Centre, School of Physics and Astronomy, University of Nottingham, Nottingham, UK; 2 NIHR Nottingham Biomedical Research Centre, Nottingham University Hospitals NHS Trust and the University of Nottingham, Nottingham, UK; 3 Nottingham Digestive Diseases Centre, School of Medicine, the University Of Nottingham, Nottingham, UK

**Keywords:** Hepatitis C, Magnetic resonance imaging, Echo-planar imaging, Sustained virologic response

## Abstract

**Methods:**

We applied multiparametric MRI to assess changes in liver composition, perfusion and blood flow in 17 patients before direct-acting antiviral (DAA) therapy and after treatment completion (within 12 weeks of last DAA tablet swallowed).

**Results:**

We observed changes in hepatic composition indicated by a reduction in both liver longitudinal relaxation time (T1, 35 ± 4 ms), transverse relaxation time (T2, 2.5 ± 0.8 ms; T2* 3.0 ± 0.7 ms), and liver perfusion (28.1 ± 19.7 ml/100 g/min) which we suggest are linked to reduced pro-inflammatory milieu, including interstitial oedema, within the liver. No changes were observed in liver or spleen blood flow, splenic perfusion, or superior mesenteric artery blood flow.

**Conclusion:**

For the first time, our study has shown that treatment of HCV with DAAs in patients with cirrhosis leads to an acute reduction in liver T1, T2 and T2* and an increase in liver perfusion measured using MR parameters. The ability of MRI to characterise changes in the angio-architecture of patients with cirrhosis after intervention in the short term will enhance our understanding of the natural history of regression of liver disease and potentially influence clinical decision algorithms.

**Key Points:**

*• DAAs have revolutionised the treatment of hepatitis C and achieve sustained virological response in over 95% of patients, even with liver cirrhosis.*

*• Currently available non-invasive measures of liver fibrosis are not accurate after HCV treatment with DAAs, this prospective single-centre study has shown that MRI can sensitively measure changes within the liver, which could reflect the reduction in inflammation with viral clearance.*

*• The ability of MRI to characterise changes in structural and haemodynamic MRI measures in the liver after intervention will enhance our understanding of the progression/regression of liver disease and could potentially influence clinical decision algorithms.*

## Introduction

Globally, chronic hepatitis C virus (HCV) infection is estimated to affect 71 million people [1]. Direct-acting antivirals (DAAs) have revolutionised HCV treatment, with sustained virological response (SVR) rates approaching 100% in compensated cirrhosis [2–4], emerging data suggesting excellent SVR in decompensated liver disease [5, 6]. Despite high SVR rates, there is an incomplete understanding of the effect of viral clearance on the liver in the context of DAA therapy. The progression or regression of fibrosis and/or portal hypertension caused by DAA therapy could have implications for each patient wider than chronic HCV management alone. Potential changes include those reflecting liver composition, including volume, inflammation and fibrosis; and hepatosplanchnic haemodynamic changes, including liver perfusion and blood flow.

Improvement in clinical outcomes following HCV eradication with treatment regimens of pegylated interferon and ribavirin is established; large cohort studies show differences in liver decompensation rates between SVR and non-SVR groups: hazard ratio (HR) 0.24 (95% CI 0.14–0.42), *p* < 0.001 [7]; HR 0.26 (95% CI 0.17–0.39), *p* < 0.001 [8]; and HR 0.15 (95% CI 0.06–0.38), *p* = 0.04 [9]. Assessment by invasive liver biopsy in HCV patients with established cirrhosis has shown regression of cirrhosis in 61% and reduction of collagen in 89% of patients at 61 months following an SVR [10]. Further studies using liver biopsy have shown cirrhosis regression rates of 46 to 75% after 3–10 years [11–14]. Although promising, regression was not ubiquitous nor studied in those with the most advanced liver disease due to the known risks of treatment with interferon and ribavirin. It remains unproven whether the regression seen was due to selection bias of those who achieved SVR, aviraemia or an immunomodulatory effect of the interferon itself [15]. Hepatic venous pressure gradient (HVPG), an invasive measure of portal hypertension, also improves with SVR [16–18]. Together, this published data builds a strong case for the concept of regression.

However, individual and invasive techniques for measuring fibrosis and portal hypertension respectively do not assess the complex pathophysiological changes associated with progression and regression of chronic liver injury. Furthermore, ethical and practical constraints limit serial liver biopsy sampling with DAA therapy. A multicentre prospective study, with paired invasive HVPG and non-invasive transient elastography (TE), demonstrated DAA therapy significantly reduced HVPG, but patients continued to have clinically significant portal hypertension and remained at risk of decompensation [19]. Currently available clinical non-invasive markers, including TE, overestimate regression compared to biopsy after SVR [19, 20]. In an era of novel antifibrotic therapy on the horizon, robust non-invasive biomarkers for use in advanced liver disease patients who receive DAA therapy to understand the structural and functional changes in the liver and stratify ongoing risk post SVR and focus interventions are required.

Non-invasive, contrast agent-free, quantitative multiparametric magnetic resonance imaging (MRI) provides the opportunity to assess liver composition (volume, fibrosis/inflammation) and haemodynamics (liver tissue perfusion, blood flow) in a single scan session (< 40 min). Longitudinal relaxation time (T_1_) of liver tissue is validated against inflammation/fibrosis on liver biopsy [21–23], and MRI measurements also closely correlate with the invasive HVPG measurement [24]. Inflammation lengthens hepatic transverse relaxation time (T_2_) in liver disease [25–27]. More recently, specific MR liver biomarkers, including liver T_1_ and liver perfusion, predicted clinical outcomes [28, 29].

Here, we collect quantitative MRI data in patients with liver cirrhosis who underwent DAA therapy from the NHS England expanded access programme [5]. This study describes the early changes in structural and haemodynamic MRI measures in the liver between baseline (pre-treatment) and follow-up to SVR (immediately post-treatment) at a 3–6-month time window following the start of DAA therapy, in patients with advanced end-stage liver disease.

## Methods

In this prospective, observational study, patients were recruited through the NHS England expanded access programme, established to prioritise treatment for patients with greatest clinical priority, including compensated and decompensated liver disease. Treatment was with sofosbuvir plus, by clinician choice, ledipasvir or daclatasvir, with or without ribavirin [5, 30]. The study received ethical approval from the NRES Committee East Midlands - Derby 1 (Research Ethics Committee reference 11/EM/0314). Once enrolled in the study, if a subject did not attend a study visit after treatment, they were sent a letter and telephoned twice by the research team and withdrawn from the study if uncontactable.

Patients underwent a detailed MRI study before DAA therapy and after treatment completion (within 12 weeks of last DAA tablet swallowed). Patients followed standard management protocols for DAA therapy and monitoring. Routine clinical information including medical history, clinical examination and laboratory values were recorded for each participant. Laboratory values were used to calculate validated scores of ALT, Fib4 [25] and APRI [26] using freely available online calculators.

## MRI measures

MRI data were acquired on a 1.5-T Philips Achieva scanner (Philips Healthcare Systems) in a single 40-min scan session using methods described in [21]. Subjects were scanned feet first supine after an overnight fast, using a body transmit and 16-element SENSEXL torso coil. The MRI protocol comprised a series of non-invasive measures to assess liver composition and haemodynamics. Multislice balanced fast field echo (bFFE) images were initially acquired in three orthogonal planes (35 slices of 1.75 × 1.75 × 7 mm^3^ resolution, single breath holds per orientation) to locate the liver and vessels of interest and to estimate liver volume.

### Liver composition

Liver T_1_, T_2_ and T_2_* were mapped in nine axial slices through the liver (field of view (FOV) 288 × 288 mm^2^, voxel size 3 × 3 × 8 mm^3^, 4-mm slice spacing). A modified respiratory-gated inversion recovery sequence with a fat-suppressed spin echo echo-planar imaging (SE-EPI) readout scheme was used to measure liver T_1_ [22, 27, 28] (inversion times for first SE-EPI image slice were 100–1000 ms in 100-ms increments). For all inversion times, SE-EPI imaging slices were collected at end expiration such that the first slice was collected at 1500 ms after the respiratory trigger with subsequent slices collected with a 65-ms temporal slice spacing. The T_1_ mapping sequence was acquired with slices collected in ascend and descend slice ordering to increase the dynamic range of inversion times. In total, 20 inversion times were acquired in < 3 min. A respiratory-gated SE-EPI sequence was used to map liver T_2_ comprising six echo times (TE = 27, 35, 42, 50, 60, 70 ms) in approximately 2 min. T_2_* mapping was collected using a multiecho fast field echo (mFFE) sequence comprising 12 echo times (TE_1_ = 5 ms, ΔTE = 2.5 ms) acquired in a ~ 17-s breath hold. T_2_ and T_2_* datasets were geometrically matched to the T_1_ dataset.

In-house software was used to create T_1_, T_2_ and T_2_* maps (MATLAB, The MathWorks Inc.). Prior to data fitting, images affected by motion (due to missing the respiratory trigger) were discarded. To create T_1_ and M_0_ maps, data at the 20 inversion times were fit using a voxel-by-voxel two-parameter fit. For T_2_ and T_2_* mapping, a voxel-by-voxel log-linear least-squares method was used to fit the echo intensities to create T_2_ and T_2_* maps. To assess the quantitative T_1_, T_2_ and T_2_* maps, a region of interest covering the liver was selected and a histogram of values within computed. A Gaussian curve was fitted to the histogram to determine the mode of the T_1_, T_2_ and T_2_* distribution within the liver; this procedure excludes regions where vessels are visible within the liver.

### Blood flow

Phase contrast (PC)-MRI assessed blood flow through vessels in the hepatic circulation (portal vein, hepatic artery) as well as vessels critically related to portal hypertension (splenic artery, right renal artery, superior mesenteric artery (SMA)) with reconstructed voxel size of 1.17 × 1.17 × 6 mm^3^ [22]. PC-MRI was performed using a single slice turbo field echo (TFE); slice was placed perpendicular to each vessel. Fifteen phases were collected across the cardiac cycle for the portal vein, 20 phases for all other vessels, with velocity encoding in the portal vein of 50 cm/s, in the hepatic, splenic, renal arteries 100 cm/s, and 140 cm/s in the SMA. Each measurement was acquired in a single < 20-s breath hold. Using Q-flow software (Philips Medical Systems), mean artery cross-sectional area (mm^2^), mean velocity (cm/s), and hence mean bulk flow (ml/s) over the cardiac cycle were calculated for each vessel.

### Liver perfusion

Respiratory-triggered flow-sensitive alternating inversion recovery arterial spin labelling (FAIR-ASL) data (288 × 288 mm^2^ field of view, 3 × 3 × 8 mm^3^ voxel, 3 sagittal slices, slice gap 5 mm) were collected with a balanced fast field echo (bFFE) readout in approximately 5 min. A base (M_0_) equilibrium scan and T_1_ map were also acquired for quantification of hepatic tissue perfusion using a kinetic model. In-house software was used to motion correct the images and perform automatic outlier rejection of images affected by movement prior to quantification of tissue perfusion [29].

## Statistical analysis

Statistical analysis was performed with *GraphPad Prism7* software. Continuous variables are expressed as mean ± standard deviation for normal data otherwise median (interquartile range), while categorical variables are reported as number of patients with (proportion of patients with) the certain characteristic.

Paired Student’s *t* test is used for comparisons between pre- and post-treatments for normally distributed data and Wilcoxon matched pairs signed-rank test when not normally distributed. All statistical analysis is Bonferroni corrected for multiple comparisons.

### Repeatability of multiparametric MRI measures

To determine between session repeatability of MRI measures, the intra-subject coefficient of variation (CoV) (defined as the standard deviation/mean) of multiparametric MRI measures was assessed. A subset of ten healthy participants (age 23–37 years, body mass index 20–26 kg/m^2^) had three scans, at least 1 week apart and within 4 weeks, at the same time of day and after an overnight fast to limit diurnal and dietary variability. This healthy participant study was approved by the University of Nottingham Ethics committee.

## Results

**Fig. 1:**
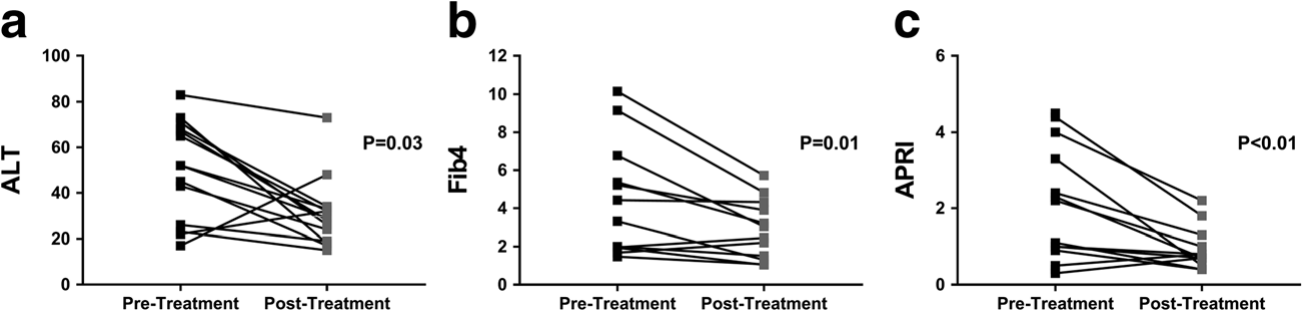
Liver function test markers of all 17 participants (16 of whom achieved SVR) pre-treatment and post-treatment. **a** A significant reduction in ALT of 54 ± 25. **b** Fib4 reduced by 1.6 ± 0.5.**c** APRI score showed a significant reduction of 31.0 ± 0.3

Seventeen HCV patients with advanced liver disease underwent DAA therapy within 1 week of their pre-treatment MRI scan (Table 1). Patients returned for their post-treatment MRI scan at a median of 22 days (3–79 days) after the last DAA taken. Study demographics are provided in Table 1. Sixteen of 17 patients (94%) achieved SVR, defined as undetectable serum viral RNA 12 weeks after treatment completion. Validated serum clinical liver markers of ALT, Fib4 and APRI were collected at pre- and post-MRI time points. The majority of patients had significantly improved liver function test scores post-treatment compared to pre-treatment (Fig. 1), with a significant group reduction in ALT, Fib4 and APRI.

**Table 1:** Pre-treatment characteristics of the 17 hepatitis C virus patients consented to this study

Demographic table	
Variable	All patients
Age, mean (SD)	53 (8)
Male (%)	14 (82%)
Transplant (%)	3 (18%)
Cirrhosis (%)	15 (88%)
MELD (IQR)	8 (7–8.25)
Compensated (%)	7 (41%)
Decompensated (%)	8 (47%)
Previous variceal haemorrhage	4 (23%)
Ascites	2 (12%)
Jaundice	2 (12%)
Diabetes (%)	2 (12%)
Body mass index median (IQR)	25.6 kg/m^2^(24.0–27.9)
HCV genotype	
1 (%)	9 (53%)
2 (%)	1 (6%)
3 (%)	7 (41%)

**Fig. 2:**
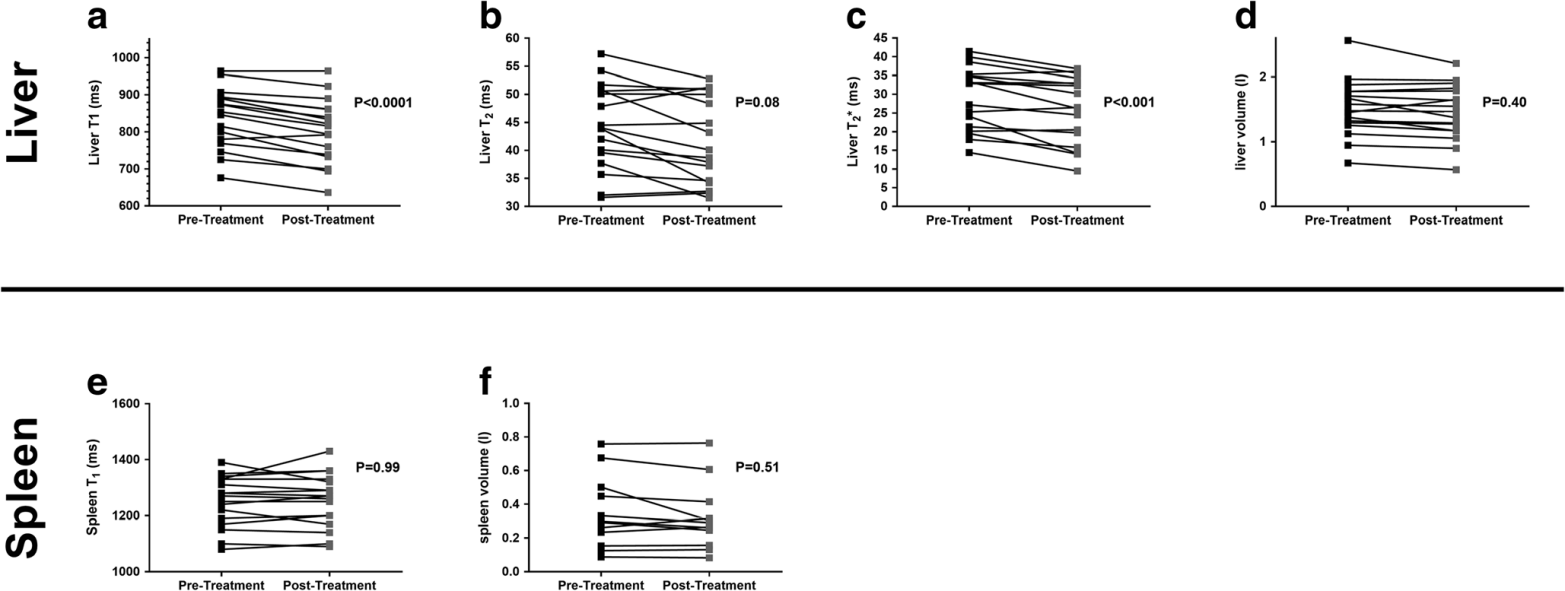
Post-treatment with DAA therapy showed (**a**) a reduction in liver T_1_ of 35 ± 4 ms, (**b**) a reduction in liver T_2_ of 2.5 ± 0.8 ms and (**c**) a reduction in liver T_2_* by 3 ± 0.7 ms. **d**–**f** No significant difference is observed in spleen T_1_, liver volume or spleen volume between pre- and post-DAA treatments

**Fig. 3:**
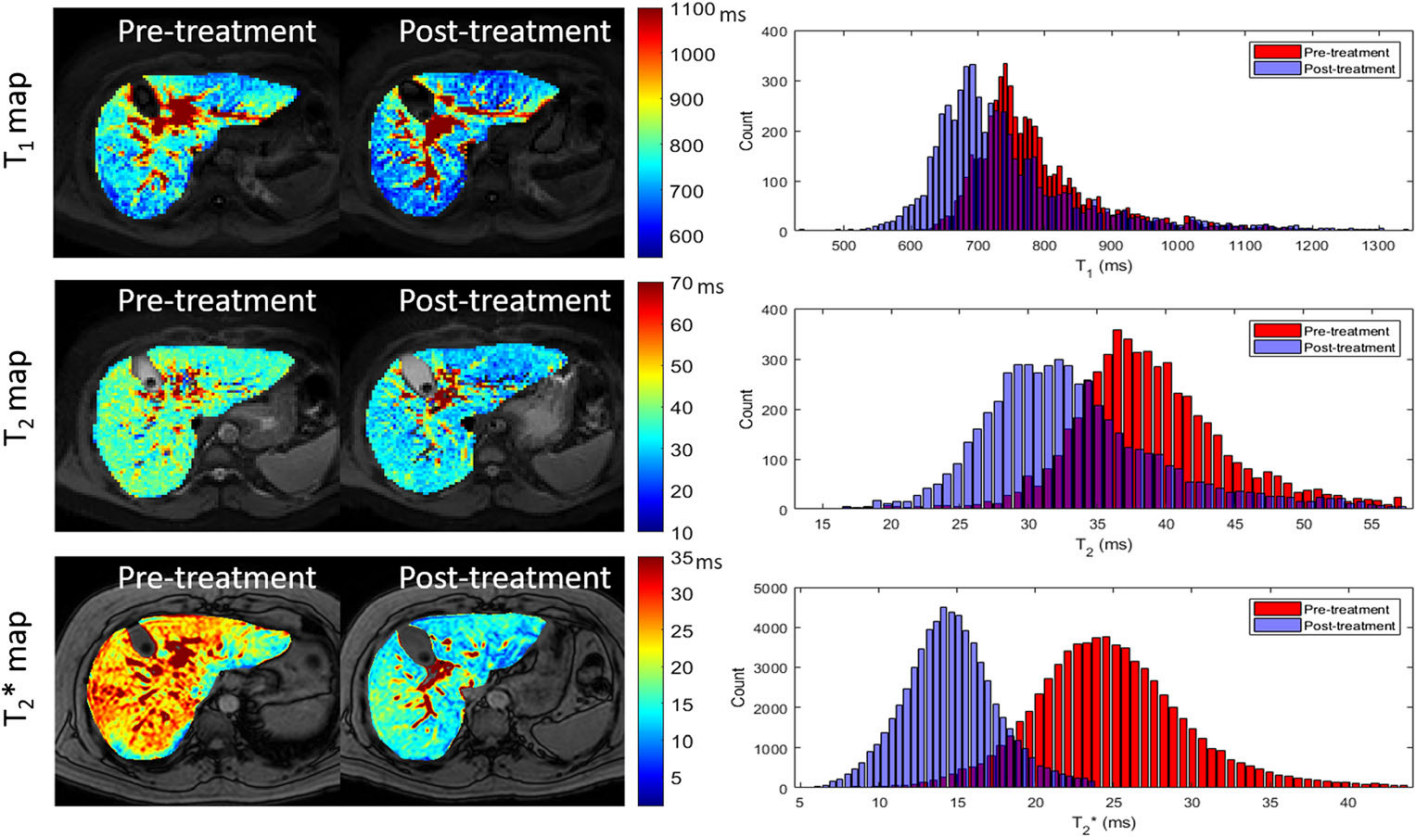
Example axial T_1_ map, T_2_ map and T_2_* map showing the liver pre- and post-DAA treatment

**Fig. 4:**
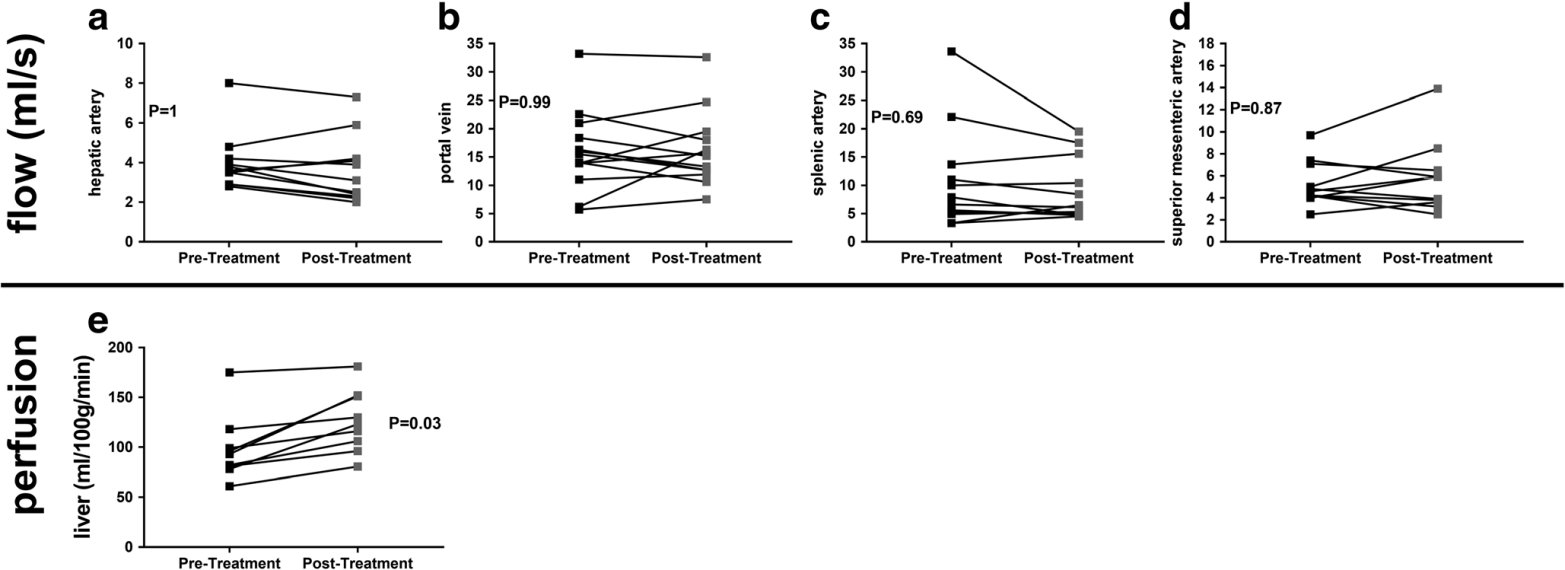
Bulk flow to the liver in the (**b**) portal vein and (**a**) hepatic artery as well as (**d**) superior mesenteric and (**c**) splenic artery flow shows no significant changes between pre- and post-DAA therapies. **e** An increase in liver perfusion of mean change 28.1 ± 19.7 ml/100 g/min is observed following DAA therapy

Table 2 shows that all MR volume and relaxometry measures had a CoV < 5%, and all haemodynamic measures < 15%, apart from hepatic artery blood flow. There were significant changes in the liver microstructure as assessed by MR relaxation times with DAA therapy, with a significant reduction in liver T_1_, T_2_ and T_2_* after treatment; however, no change was observed in splenic T_1_ (Fig. 2). Figure 3 shows the example of liver T_1_, T_2_ and T_2_* maps pre- and post-DAA therapy. No significant differences were observed in liver or spleen volume. No significant changes were observed in any blood flow measure (hepatic artery, splenic artery, superior mesenteric artery or portal vein); however, there was an increase in liver perfusion following DAA therapy (Fig. 4). Paired perfusion data is presented for *n* = 9 participants, all of whom achieved SVR. The remaining subjects had inadequate paired data due to insufficient anatomical matching between visits.

**Table 2:** Coefficient of variance of MRI measures

MRI Measure	CoV (%)
Liver volume	4.6
Liver T_1_	1.5
Liver T_2_	4.3
Liver T2*	3.7
Portal vein flow	13.6
Hepatic artery flow	22.7
Liver perfusion	12
Spleen volume	5.2
Spleen T_1_	1.8
Splenic artery flow	11
SMA flow	7.6

## Discussion

Using multiparametric MRI in patients with HCV-related cirrhosis pre- and post-DAA therapy, we have demonstrated significant changes in the liver composition (T_1_, T_2_ and T_2_*) and haemodynamics over a short time period following clearance of HCV infection. We did not observe any changes in bulk hepatic or splanchnic blood flow in the short time frame between MRI scans.

To our knowledge, this is the first study to document changes in MR parameters following DAA therapy. Few previous studies have assessed the effect of HCV treatment on MRI measures. One previous study assessed the effect of HCV treatment (pegylated interferon, ribavirin, telaprevir) on liver diffusion, demonstrating reduced liver apparent diffusion coefficient suggested to be associated with ultrastructural changes such as cell necrosis/apoptosis and inflammatory cell infiltration [31]. A recent study showed a small increase in liver volume following antiviral treatment, which was larger in patients with SVR [31], interpreted to indicate liver regeneration and/or recovery and reduced fibrotic load of the liver.

The strengths of this current study are the prospective recruitment and phenotyping of the patients. The quantitative MRI parameters have previously been validated against “gold standard” measures including liver biopsy [19, 20] and HVPG [22]. More recently, specific MR liver biomarkers, including liver T_1_, liver perfusion and haemodynamic measures, were associated with clinical outcomes in independent cohorts of patients [28, 29]. Liver T_1_ acquisition and analysis have been shown to be highly repeatable [19], with an intra-subject CoV < 1.8% and a low inter- and intra-observer variability with intra-class correlation coefficient > 0.99 [22]. Here, we demonstrate the intra-subject variability in MR relaxation time is low, with a CoV of 1.5, 4.3 and 3.7% for liver T_1_, T_2_ and T_2_* respectively, considerably lower than inter-subject variability. Capturing data pre- and post-treatment enables direct intra-individual comparisons, strengthening the validity of the data, since each subject is their own control. We show that in response to DAA therapy, the reduction in T_1_ is more significant compared to that of T_2_ and T_2_*; this could be attributed to the smaller CoV. However, there is also variability within the literature in terms of a T_2_ change, with pre-clinical models of liver fibrosis shown to result in an increase as well as decrease in T_2_ [32]. It is hypothesised that increased T_2_ is related to hepatic inflammation associated with the development of fibrosis or the proliferation of small biliary ducts in models of bile duct ligation [27].

The limitations of this study are the small sample size from a single UK centre, with some variation in patient disease severity. Due to practical constraints, we were unable to perform parallel invasive assessments of liver biopsy or HVPG. The timing of the MRI scans in close proximity to drug therapy enabled characterisation of changes at an early time point in subjects who achieve SVR at a later time point.

The observation that only few specific MR parameters changed in the study time period is relevant. Notwithstanding the possibility of type 1 and type 2 errors, the lack of significant changes in liver bulk blood flow potentially indicates the natural history of “regression”. Reversal of fibrotic and vascular networks, which of course may be incomplete [33], is thought to occur over years. To date, there is limited data showing histological changes associated with DAA treatment in HIV coinfection [34] and post-transplant populations [35], both demonstrating a significant reduction in necroinflammation with SVR. It is widely recognised that both TE and serum markers of fibrosis are influenced by necroinflammation [20, 36–38]. Moreover, short-term studies have shown that TE dynamically changes during treatment with DAA therapy, and long-term studies have shown that both TE and serum fibrosis markers may overestimate regression in the long term [19, 33].

Pegylated interferon and ribavirin ameliorate portal hypertension in patients with HCV monoinfection [16–18] and HIV/HCV coinfection [39]. Multiple studies similarly show that DAA treatment results in statistically significant reductions in HVPG [19, 34, 40, 41] but interestingly may not alter clinically significant portal hypertension [19].

We speculate our key findings of reduced liver T_1_, T_2_ and T_2_* and increased liver perfusion are linked by a reduction in the pro-inflammatory milieu within the liver, including interstitial oedema, aligned with a reduction in serum ALT. We hypothesise that DAA treatment reduces necroinflammation which may improve liver function over a longer period of time [30] and can be a treatment for portal hypertension provided treatment in the early stage of portal hypertension [19], and we believe this also underlies the reported change in TE [20]. A reduction in necroinflammation on liver biopsy in the short term, even when associated with a short duration of viral suppression using interferon-based treatment, has a positive impact on future clinical outcomes at 6 years and fibrosis regression [42]. Chronic inflammation might be expected to increase perfusion but is unknown in the context of advanced liver disease. A recent MRI study showed reduced perfusion is associated with progressive liver disease and linked to adverse outcomes [29], and CT has shown worsening perfusion with progressive fibrosis in HCV [43]. This is consistent with our finding of a significant increase in liver perfusion with the likely acute resolution of chronic necroinflammation after DAA treatment in advanced liver disease caused by HCV.

A multimodal technique including MRI that captures how the different aspects of liver composition, perfusion and blood flow change over time could provide additional confidence for clinical decision-making. In addition, robust non-invasive tests that are specific to the liver would be valuable to drug development for antifibrotic compounds as they can be repeated at multiple time points to evaluate drug efficacy. MRI has the potential to be a key non-invasive tool to evaluate the efficacy of interventions in chronic liver disease and stratify patients according to the potential clinical outcomes.

In summary, for the first time, our MRI study has shown that treatment of HCV with DAAs in patients with cirrhosis leads to an acute reduction in liver T_1_, T_2_ and T_2_* and increase in liver perfusion measured. The ability of MRI to characterise changes in the angio-architecture of patients with cirrhosis after intervention at such short intervals will enhance our understanding of the progression/regression of chronic liver disease and potentially assist clinical decision-making. The sensitivity of these MR measures should be exploited to accelerate early phase of clinical development of novel antifibrotic agents.

In future work, our intention is to use quantitative MRI measures to observe the long-term effects of DAA therapy on liver composition, perfusion and surrounding haemodynamics to characterise the extent of fibrosis regression, vascular remodelling and reduction in portal hypertension that may occur after DAA therapy. Furthermore, we aim to assess whether MRI changes correspond to, or are predictive of, histological regression of fibrosis, as described in long-term studies with interferon and ribavirin [10, 44, 45].

## Abbreviations

DAAs: Direct-acting antiviral treatments
HCV: Hepatitis C virus
HVPG: Hepatic venous portal gradient
NHS: National Health Service
SVR: Sustained virological response
